# Non-Equilibrium Modeling of Concentration-Driven processes with Constant Chemical Potential Molecular Dynamics Simulations

**DOI:** 10.1021/acs.accounts.2c00811

**Published:** 2023-04-25

**Authors:** Tarak Karmakar, Aaron R. Finney, Matteo Salvalaglio, A. Ozgur Yazaydin, Claudio Perego

**Affiliations:** † Department of Chemistry, Indian Institute of Technology, Delhi, Hauz Khas, New Delhi 110016, India; ‡ Thomas Young Centre and Department of Chemical Engineering, University College London, Torrington Place, London WC1E 7JE, United Kingdom; ¶ Department of Innovative Technologies, University of Applied Sciences and Arts of Southern Switzerland, Polo Universitario Lugano, via la Santa 1, 6962 Lugano-Viganello, Switzerland

## Abstract

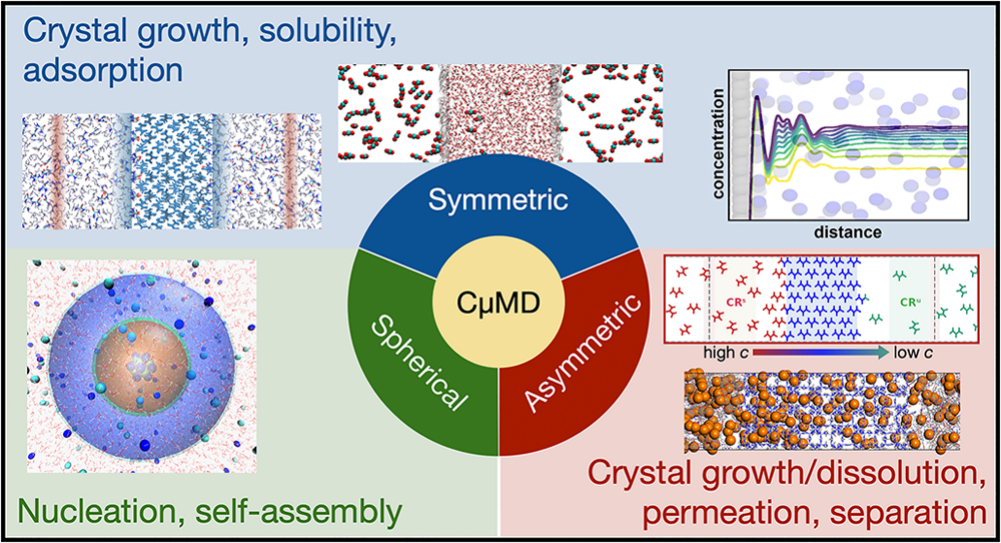

Concentration-driven processes in solution, i.e., phenomena that are sustained by persistent concentration gradients, such as crystallization and surface adsorption, are fundamental chemical processes. Understanding such phenomena is crucial for countless applications, from pharmaceuticals to biotechnology. Molecular dynamics (MD), both in- and out-of-equilibrium, plays an essential role in the current understanding of concentration-driven processes. Computational costs, however, impose drastic limitations on the accessible scale of simulated systems, hampering the effective study of such phenomena. In particular, due to these size limitations, closed system MD of concentration-driven processes is affected by solution depletion/enrichment that unavoidably impacts the dynamics of the chemical phenomena under study. As a notable example, in simulations of crystallization from solution, the transfer of monomers between the liquid and crystal phases results in a gradual depletion/enrichment of solution concentration, altering the driving force for phase transition. In contrast, this effect is negligible in experiments, given the macroscopic size of the solution volume. Because of these limitations, accurate MD characterization of concentration-driven phenomena has proven to be a long-standing simulation challenge. While disparate equilibrium and nonequilibrium simulation strategies have been proposed to address the study of such processes, the methodologies are in continuous development.

In this context, a novel simulation technique named constant chemical potential molecular dynamics (CμMD) was recently proposed. CμMD employs properly designed, concentration-dependent external forces that regulate the flux of solute species between selected subregions of the simulation volume. This enables simulations of systems under a constant chemical drive in an efficient and straightforward way. The CμMD scheme was originally applied to the case of crystal growth from solution and then extended to the simulation of various physicochemical processes, resulting in new variants of the method. This Account illustrates the CμMD method and the key advances enabled by it in the framework of *in silico* chemistry. We review results obtained in crystallization studies, where CμMD allows growth rate calculations and equilibrium shape predictions, and in adsorption studies, where adsorption thermodynamics on porous or solid surfaces was correctly characterized via CμMD. Furthermore, we will discuss the application of CμMD variants to simulate permeation through porous materials, solution separation, and nucleation upon fixed concentration gradients. While presenting the numerous applications of the method, we provide an original and comprehensive assessment of concentration-driven simulations using CμMD. To this end, we also shed light on the theoretical and technical foundations of CμMD, underlining the novelty and specificity of the method with respect to existing techniques while stressing its current limitations. Overall, the application of CμMD to a diverse range of fields provides new insight into many physicochemical processes, the *in silico* study of which has been hitherto limited by finite-size effects. In this context, CμMD stands out as a general-purpose method that promises to be an invaluable simulation tool for studying molecular-scale concentration-driven phenomena.

## Key References

- PeregoC.; SalvalaglioM.; ParrinelloM.Molecular dynamics simulations of solutions at constant chemical potential. J. Chem. Phys.2015, 142, 14411310.1063/1.491720025877568.^1^ This article introduces the CμMD method, reporting its application to the test case of urea crystallization from an aqueous solution.
- OzcanA.; PeregoC.; SalvalaglioM.; ParrinelloM.; YazaydinO.Concentration gradient driven molecular dynamics: a new method for simulations of membrane permeation and separation. Chemical Science2017, 8, 3858–386510.1039/C6SC04978H28966778PMC5578366.^2^ This article reports the first application of asymmetric CμMD to study the permeation of organic compounds through a zeolitic imidazolate framework-8 membrane.
- KarmakarT.; PiaggiP. M.; ParrinelloM.Molecular dynamics simulations of crystal nucleation from solution at constant chemical potential. J. Chem. Theory Comput.2019, 15, 6923–693010.1021/acs.jctc.9b0079531657927(3) This article presents the CμMD method in its spherical implementation, applied to the homogeneous nucleation of sodium chloride from an aqueous solution.
- FinneyA. R.; McPhersonI. J.; UnwinP. R.; SalvalaglioM.Electrochemistry, ion adsorption and dynamics in the double layer: a study of NaCl(aq) on graphite. Chemical Science2021, 12, 11166–1118010.1039/D1SC02289J34522314PMC8386640(4) This article presents symmetric CμMD to investigate the adsorption of ions at carbon surfaces with insight into the concentration dependence of solution electrochemistry and activity.

## 1 Introduction

Molecular dynamics (MD) simulation is a useful technique for investigating in microscopic detail physicochemical and biological processes where imposing the thermodynamic conditions that control a process of interest, including temperature and pressure, is essential. In this Account, we address the key challenge of simulating out-of-equilibrium concentration-driven processes, i.e., physicochemical processes that are sustained by solution concentration gradients, subject to persistent differences in chemical potential and to the steady transfer of molecules. Whereas the simulation of these processes is of considerable interest for the study of, e.g., crystallization, transport through porous media, biomolecular condensation, it is less than straightforward. This is mainly because out-of-equilibrium simulations cannot exploit equilibrium statistical mechanics and must rely on the complexity and lower predictive capabilities of nonequilibrium theories.^5,6^ In addition, these simulations are most often affected by finite-size artifacts; typical MD simulations contain up to ∼10^7^ atoms/particles, with periodic boundary conditions (PBCs) that remove surface effects. This limited size is often inadequate to study concentration-driven processes, as a constant thermodynamic force that “feeds” the process of interest is required. For example, during crystallization, solute molecules are drawn from the solution to the crystallite. In standard closed-system simulations with a fixed number of molecules, this results in solute depletion and a reduction in crystal growth rates due to a decreasing supersaturation. Therefore, comparisons with experiments, where the bulk composition is almost constant over μs time scales, are challenging at best. Whereas theoretical corrections to such finite-size effects have been proposed,^7−15^*ad hoc* simulation techniques are crucial for this purpose.

Overcoming finite-size limitations ideally requires open-system simulation, a crucial challenge in molecular modeling.^16^ The core feature of open-system molecular simulation is the free exchange of molecules between an explicitly modeled finite-sized region of the system, which hosts the molecular process of interest, and an external reservoir. This strategy is typically employed in equilibrium molecular simulations to sample the grand canonical (GC) ensemble, where the total number of molecules varies and the chemical potential μ is constant. This is the case in simulation methods such as GC-MD,^17−20^ that introduced variable-particle Hamiltonians, GC-Monte Carlo (GC-MC),^21−23^ or hybrid GC schemes,^20,24^ coupling MD or MC engines with stochastic particle insertion/deletion algorithms. While variable-particle MD schemes are still not broadly exploited, particle insertion methods have been applied more extensively, also for *in silico* chemical potential calculation^25^ and in nonequilibrium simulations (see below). Nonetheless, when simulating condensed matter systems, the inevitable very low probabilities for particle insertion can be problematic.^26^ This issue can be addressed by enhancing the cavity location,^27^ using nonequilibrium MC steps,^28^ or introducing extra dimensionalities.^29^ Recently, the effect of open boundaries was also implemented by employing a machine-learning strategy based on neural networks.^30^

Given the difficulties in open-system simulation, alternatives were also proposed, for example, by combining carefully prepared closed simulations, representing subsequent steps along a proper reaction coordinate, to maintain the solution concentration as the chemical process, e.g., nucleation, progresses.^31^

A widespread closed-system strategy to reduce finite-size effects is the multiple resolution approach in which a region of interest is described with high resolution, typically atomistic, and the remaining system is described using a lower-resolution (coarse-grained) model, which acts as a molecule reservoir for the system. While this allows the simulation of relatively large volumes,^32−34^ special care is required to couple these two regions. To this aim, different strategies were developed, including AdResS^35^ and H-AdResS,^36−38^ that have been successfully applied to explore a broad class of systems and phenomena, including the dynamics of polymers and biomolecules in water. Multiple resolution methods were recently coupled with insertion/deletion algorithms as well, exploiting the simplified reservoir description to overcome the low probability issues.^39,40^

Whereas the aforementioned schemes were typically proposed for equilibrium GC simulation, the possibility to supply/remove molecules to/from a region of interest makes these methods suitable for studying concentration-driven, out-of-equilibrium processes.^16^ For example, MC moves are adopted to insert/remove particles in the dual-control volume GCMD^41,42^ and nonequilibrium chemical potential gradient-based methods^43,44^ in which a chemical potential gradient is generated between the “source” (high-concentration) and “sink” (low-concentration) regions of the simulation cell. These methods have been successfully applied to study gas diffusion and separation,^45−48^ but in the presence of condensed matter, they still suffer from low particle insertion probabilities. In closed systems, concentration-driven simulations can also be achieved by applying external forces to establish molecular flows. This field-driven nonequilibrium approach was employed to study osmosis,^49,50^ diffusiophoresis,^51^ and transport in porous media.^52,53^ This extends to studies of crystallization that exploit external forces or local temperature control to establish a molecular flow facilitated by the simultaneous dissolution and growth of a crystal slab.^54,55^

In this Account, we focus on the constant chemical potential molecular dynamics (CμMD) technique,^1^ which allows us to model processes evolving out of equilibrium under the effect of concentration gradients. CμMD establishes steady-state concentration gradients in a closed system by locally controlling the concentration of the species that populate it. This is attained by a proper subdivision of the simulation volume and by the application of concentration-dependent forces, which regulate the molecular diffusion throughout the system without directly perturbing the dynamics of the process of interest. Here we discuss the theoretical foundations of CμMD and present results from its application to a wide range of systems, describing current limitations and potential developments of the method.

## 2 Constant Chemical Potential Molecular Dynamics (CμMD)

When a fluid phase is in contact with a phase boundary acting either as a “sink” or “source” for component *i*, mass transfer to/from the interphase boundary is established. By regulating the composition of the fluid phase, CμMD controls the chemical potential difference of *i* across the phase boundary, i.e., its mass-transfer driving force. For instance, when the sink/source is a crystal at constant temperature *T* and pressure *P*, the chemical potential difference is^56^
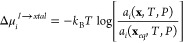
1In a system with *N* components, the equilibrium activity *a*_*i*_(**x**_*eq*_, *T*, *P*) is constant, so controlling *N* – 1 concentrations keeps the molar fraction vector **x** = {*x*_*i*_}_*i*=1_^*N*^ (and with it *a*_*i*_) constant, consequently controlling (further details in the Supporting Information).^57^

### 2.1 Symmetric Scheme

**Figure 1:**
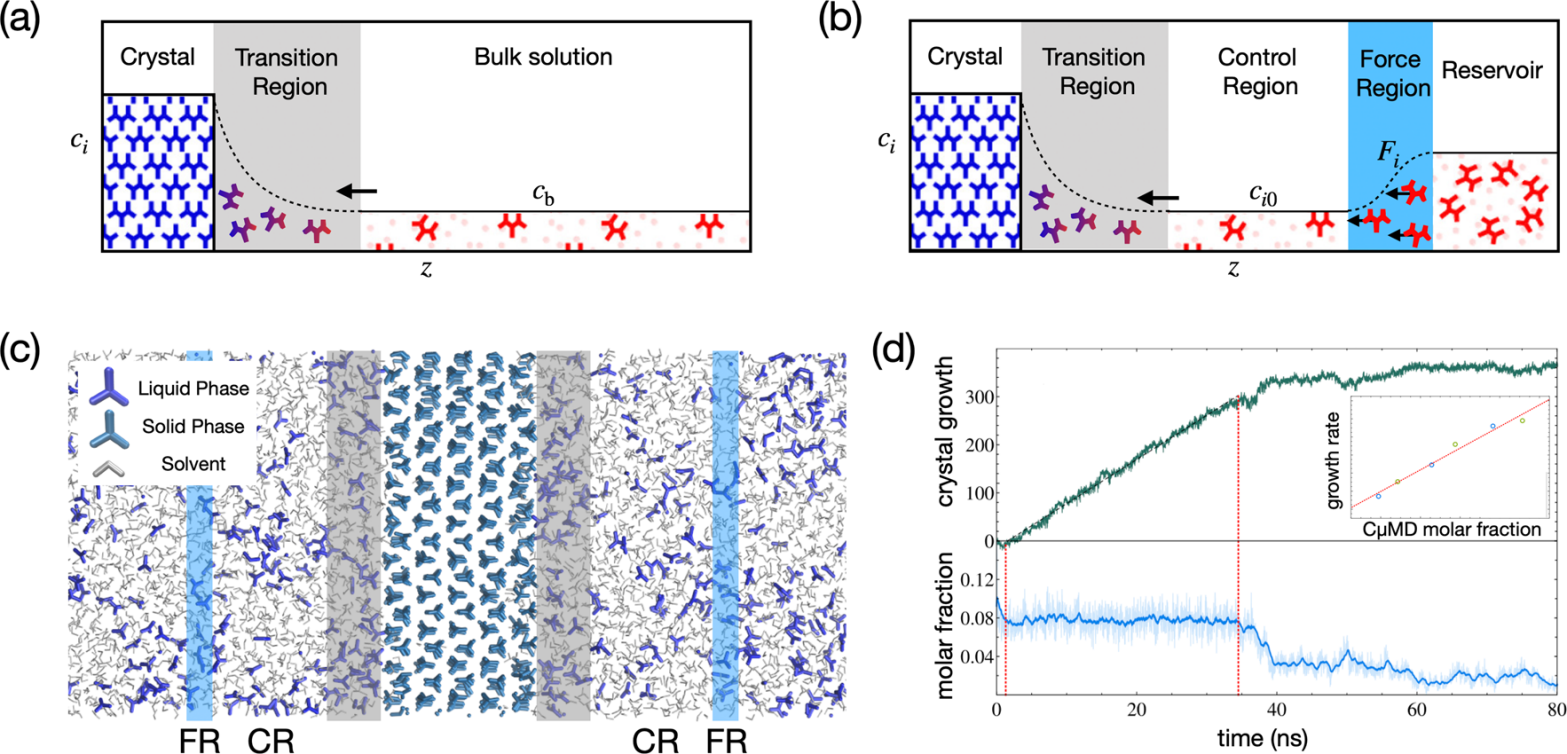
Scheme of the CμMD method. (a) Solute concentration profile *c*_*i*_ of a planar (urea) crystal in solution. (b) CμMD action and definition of different regions of the solution volume. (See the text.) (c) Symmetric CμMD scheme applied to a urea crystal surrounded by aqueous urea solution. As in (b), the TR is highlighted in gray and the FR is in blue. (d) Urea crystal growth simulated via CμMD. The crystal size variation (molecule number, top) and molar fraction in the CR (bottom) are shown vs MD time. Constant molar fraction is enforced for several ns, enabling the growth rate estimate. This demonstrates the linear growth rate dependence on the solution composition (inset).^1^ Adapted with permission from ref (1) (copyright 2015, AIP).

CμMD was originally developed to simulate urea crystal growth in solutions with constant chemical potential (Figure 1).^1^ The urea concentration, *c*_*i*_, was assumed to be homogeneous throughout the liquid volume, except in the region close to the crystallite, where the phase transition perturbs *c*_*i*_ from the bulk value *c*_b_, creating a concentration gradient perpendicular to the crystal–solution interface (Figure 1a). As crystallization proceeds, the phase boundary shifts and urea is depleted from the solution, lowering *c*_b_. The interface, therefore, acts as a “sink” for solute species. To maintain the driving force for crystallization, namely, the solution supersaturation, extra solute molecules must be supplied so that  is constant (eq 1). Alternatively, if the urea crystal dissolves in an undersaturated solution, then it acts as a “source” of molecules. In this case, the driving force for dissolution can be kept constant by removing the additional solute.

CμMD applies external forces to control the composition of the solution phase in selected regions surrounding the phenomenon of interest. This is schematized in Figure 1b, which shows the solute concentration profile *c*_*i*_(*z*) enforced by CμMD in the presence of an interface region. As in Figure 1a, a transition region (TR) delimits the solution layer adjacent to the interface, where *c*_*i*_ is perturbed by the sink/source effect of the phase boundary. Outside the TR, *c*_*i*_(*z*) converges to the bulk value *c*_b_. CμMD partitions the solution bulk into (i) the control region (CR), where *c*_*i*_ is kept constant as crystallization/dissolution proceeds, (ii) the force region (FR), where the external controlling forces act, and (iii) the remaining molecule reservoir (Figure 1b). In planar crystal growth, two interfaces are simulated, as shown in Figure 1c. The instantaneous concentration of species *i* inside the CR is
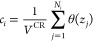
2where *V*^CR^ is the CR volume, *N* is the total number of solute particles (*j*), and *θ*(*z*) indicates if the position of the molecule *z* is inside (*θ* = 1) or outside (*θ* = 0) the CR. For practical reasons, e.g., reducing fluctuations in *c*, *θ* is defined as a continuous, differentiable switching function.^1^ To regulate the molecular flow toward the CR, enforcing a target *c*_*i*_ (*c*_*i*0_), CμMD applies a harmonic-like force
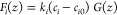
3where *k*_*i*_ is a properly chosen force constant and *G*(*z*) is a bell-shaped function that localizes *F*_*i*_(*z*) in the FR. In planar geometry, *G* is chosen as
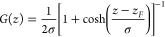
4where σ determines the FR width. When the CR concentration *c*_*i*_ deviates from the desired value *c*_0_, as a result of crystallization/dissolution, the solute molecules in the FR are accelerated to balance this deviation. The user selects the location of the CRs and FRs, which species are subject to *F*_*i*_, the force constants *k*_*i*_, and the target concentrations *c*_*i*0_. This scheme constitutes the core of the CμMD method, which is then conveniently adapted using system-dependent precautions for the concentration-driven phenomenon of interest. We underline that the CμMD external forces interfere with the equilibrium ensemble sampled in the entire simulation box. However, we assume that this interference does not affect the dynamics (and the desired sampling) in the active region where the process of interest takes place. To support this assumption, in the study of urea crystallization at constant chemical potential,^1^ geometry adaptivity was implemented: the crystal interface is located on-the-fly by monitoring the solvent distribution, and the positions of CR and FR are regulated accordingly, maintaining a fixed distance from the phase boundary. Moreover, the CR and FR geometries were appropriately set after thoroughly studying the solute and solvent concentration profiles with and without *F*_*i*_, aiming at decoupling the active region from the FR and reservoir volume. Since the simulated system is finite, this decoupling holds temporarily, but this is often sufficient to estimate crystallization/dissolution rates at constant composition (Figure 1d). In general, to minimize any direct influence of *F*_*i*_ on the “active” region, preliminary studies to determine the optimal control (such as in ref (1)) are required. This procedure can be more or less demanding, according to the requirements of the simulation (for example, if rigorous sampling is needed for a long time) and depending on the properties of the solution (such as diffusivity). Overall, this means that the CμMD method cannot be applied in a “black box” fashion before having attained sufficient knowledge of the simulated system. However, this preparatory stage typically does not exceed the standard preliminary system assessment that MD simulations normally require (for example, at the equilibration stage), involving relatively short simulation steps.

#### 2.1.1 Crystallization and Solubility

As mentioned above, CμMD was first applied to urea crystallization,^1^ investigating both the rapidly (001) and slowly growing (110) urea crystal faces.^7,8^ CμMD allows estimating growth rates at constant supersaturation, thus revealing crystallization trends (Figure 1d) and enabling comparisons to experiments. In another CμMD application, crystal growth in binary Lennard-Jones mixtures was simulated,^58^ highlighting how CμMD effectively controls the local composition of specific species near a growing crystal surface. The method was further utilized to investigate solvent effects on the crystal morphologies of an antituberculosis drug, isoniazid (INH), in alcohol solvents.^59^ The solvent’s role in crystal growth was investigated here, fixing the solution supersaturation and estimating the relative growth rates of the two INH surfaces. This allowed the prediction of the equilibrium, solvent-specific crystal geometry. Recently, CμMD was used to calculate relative growth rates for the (011) and (010) faces of glycine crystals in aqueous solutions and investigate the impact of ions on the crystal morphology.^60^

CμMD was also combined with well-tempered metadynamics^61^ (WTMetaD) to predict supersaturation-dependent naphthalene crystal shapes in ethanol.^62^ WTMetaD enhances the sampling of crystallization/dissolution events over simulation time scales by driving the system along carefully selected reaction variables, the collective variables (CV). Here, for example, a CV indicating the surface-layer crystallinity of solutes was used so that WTMetaD could efficiently sample the growth and dissolution of a new layer on the crystal. This allowed the characterization of the growth rates of the 201̅, 11̅0, and 001̅ faces of naphthalene. During enhanced growth/dissolution, concentration fluctuations were controlled by CμMD, entailing the prediction of the steady-state crystal shape. The combined WTMetaD-CμMD approach has been further applied to investigate kink-site growth at different solution concentrations, from undersaturated to supersaturated regimes.^63,64^ Herein, CμMD controlled the solution concentration during the calculation of free-energy differences between the crystalline and dissolved solute states.

CμMD was also successfully applied to solubility calculations. Coupling the method with WTMetaD enabled the prediction of the equilibrium solubility of molecular solids and salts.^63,64^ A standard symmetric CμMD scheme was instead sufficient for computing gaseous CO_2_ solubility in liquid water. Ansari et al. observed that standard MD of gas solubility resulted in an ∼10–20% vapor pressure depletion. CμMD alleviated this issue, providing a calculated CO_2_ solubility (*m*_CO_2__^(calc)^ = 0.0135) in excellent agreement with the experimental solubility (*m*_CO_2__^(exp)^ = 0.0140).^65^

#### 2.1.2 Adsorption

The adsorption of fluids in porous materials is often simulated using GCMC. Particle insertion/removal in a pore region from/to a reservoir at fixed chemical potential is used here to establish the equilibrium distribution of fluids in the pores.^23,66^ While, GCMC does not provide information on the diffusion of molecules from the bulk phase into the pores, CμMD can provide this mechanistic insight.

Loganathan et al.^67^ applied CμMD to investigate CO_2_ and CH_4_ adsorption in clay mineral slit pores. They created constant composition reservoirs of equimolar CH_4_/CO_2_ gas mixtures in control volumes external to Na-montmorillonite slit pores with variable thicknesses. Simulations indicate that both the basal pore surfaces and broken edge surfaces of montmorillonite favor CO_2_ over CH_4_ adsorption, and near the aforementioned surfaces, CO_2_ readily displaces CH_4_. The preference for CO_2_ was due to its favorable interaction with the surfaces out to ∼20 Å. In larger pore thicknesses, however, the fluid composition far from the surfaces approached that in the external control volumes. Simulations were later extended to consider Illite slit pores with varying thicknesses, investigating the role of K^+^ and Na^+^ cations on the adsorption of CO_2_ and CH_4_ from an equimolar mixture.^68^ Here, basal surfaces interact more favorably with CO_2_, especially when K^+^ is the exchangeable cation, and the near-surface CO_2_ structures were more stable than in the Na^+^ case. In contrast, the protonated edge surfaces were shown to favor CH_4_ adsorption.

**Figure 2:**
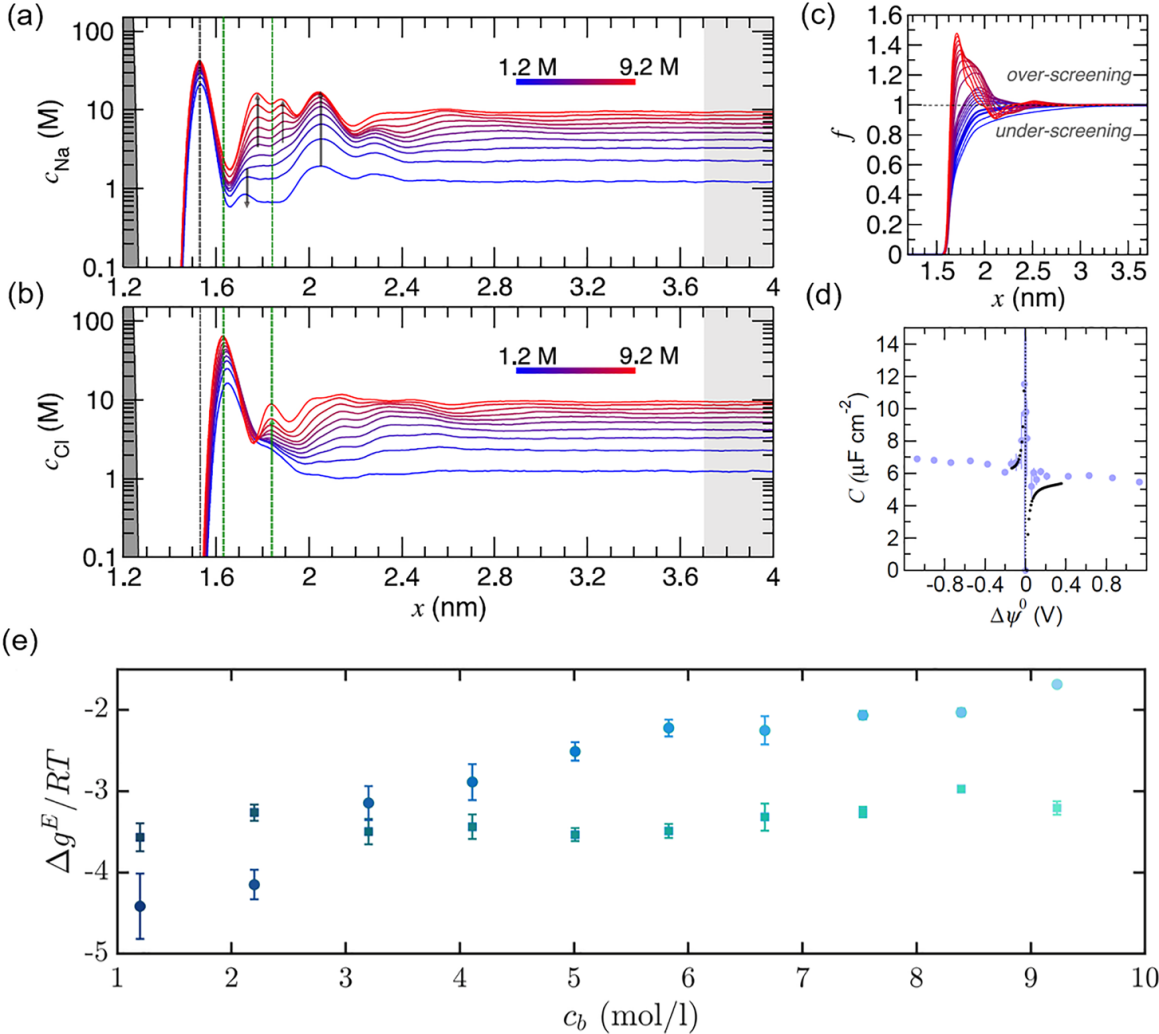
Sodium (a) and chloride (b) solution concentration profiles perpendicular to a graphite basal plane (gray bar at *x* ≈ 1.2 nm). Arrows and dashed lines indicate changes to the profiles as the bulk solution concentration changes, highlighted by the color scale. The rightmost gray region indicates the CμMD CR. (c) Screening factor, *f*, identifying how the asymmetric charge screening is affected by the bulk concentration. (d) Integral capacitance was measured in experiments (black points) and evaluated in CμMD simulations (blue points) at 1 M bulk solution concentration. (e) Excess free energy, *Δg*^*E*^, for ions at the interface vs bulk concentration.^57^ The squares and circles represent Cl^–^ and Na^+^, respectively. (a–d) Adapted with permission from ref (4) (copyright 2021, the authors). Published by the Royal Society of Chemistry under a Creative Commons Attribution 3.0 Unported License. (e) Reproduced from ref (57) (copyright 2022, Elsevier).

CμMD was recently adopted by Finney et al. to investigate the adsorption/depletion of Na^+^ and Cl^–^ ions at graphite basal plane surfaces over a wide range of bulk aqueous solution concentrations while maintaining a constant thermodynamic driving force for adsorption.^4^ Here, favorable interactions between Na^+^ and carbon π-orbital electrons (implicitly captured by the classical force field fitted to electronic structure calculations) led to an accumulation of cations at the graphite/solution interface (Figure 2a). The asymmetric ion adsorption modified the electric potential drop on the solution side of the so-called double layer (DL) as a function of concentration, consistent with experimental measurements. At the highest concentrations, the adsorption of cations also led to the accumulation of anions in a partially saturated, multilayered interface region that emerges due to ion crowding (Figure 2a,b). The coordinate *x* (perpendicular to the basal surface) where ion concentrations converge marks the outermost DL edge, the analysis of which showed an inflection point in DL size as a function of bulk concentration, in contrast to mean-field predictions. This is due to overscreening and ion crowding in the DL at high concentrations, as highlighted by the electrical screening factor (*f*(*x*′) = ∫_0_^*x*′^*c*_Cl_(*x*) d*x*/∫_0_^*x*′^*c*_Na_(*x*) d*x*) in Figure 2c, which shows deviations from the predicted monotonically increasing *f* as the bulk concentration increases.

CμMD was essential to avoid ion depletion in the bulk and maintain electroneutral bulk solutions, which would otherwise result in unphysical electrochemical artifacts when computing the capacitance of the graphite basal surface. Finney et al. varied the surface charge density (σ) of graphite in ∼40 simulations from ±0.04 to ±1 *e* nm^–3^.^4^ The resulting total integral capacitance was evaluated according to *C* = σ/*Δψ*^0^, where *Δψ*^0^ is the potential of zero charge shifted potential drop. Excellent agreement between *C* evaluated in simulations and measured in experiments is apparent in Figure 2d. From these results and the analysis of the differential capacitance, Finney et al. were able to re-evaluate the long-held assumption that ion accumulation in the DL represents a minor contribution to the capacitance of graphite and demonstrated that changes in electrochemical properties are related to changes in the screening factor across the interface.

The equilibrium between solutions in the DL and the bulk (kept at controlled composition) facilitated the evaluation of the excess ion adsorption free energies (*Δg*^*E*^), obtained using Maxwell–Stefan diffusive mass transfer to describe ion fluxes.^57^ This description of *Δg*^*E*^ accounts explicitly for the electric fields induced by the mobile charges in the double layer. The asymmetric driving force for ion surface adsorption is shown in Figure 2e by differences in *Δg*^*E*^ for Na^+^ and Cl^–^, which indicate a clear concentration dependence. Indeed, a change in the most negative *Δg*^*E*^ occurs at 3 to 4 mol/L, with anion adsorption around 4 kJ/mol more favorable than cation adsorption at the highest sampled concentrations due to changes in DL structure and charge screening. Small fluctuations in the CR concentrations, independent of the target, ensured accurate identification of the concentration range where this transition is observed.

### 2.2 Asymmetric Scheme

The symmetric CμMD scheme has been successfully applied to study crystallization, solubility, and surface adsorption in solution.^1,4,62,65,69^ However, symmetric CμMD is limited by the finite capacity of the reservoir in supplying or consuming species whose concentration is controlled in the CR. As a result, the constant gradient regime lasts as long as the reservoir population is sufficient to maintain the CR concentration (Figure 1d).^1^ Afterward, finite-size effects will interfere with the dynamics of the CRs, affecting the quantitative output of CμMD. This finite-reservoir limitation is avoided in the asymmetric variant of the CμMD, first applied to the study of fluid permeation through porous membranes^2^ and, subsequently, to the simultaneous investigation of the growth and dissolution of crystals in solution.^70^

#### 2.2.1 Permeation Studies

**Figure 3:**
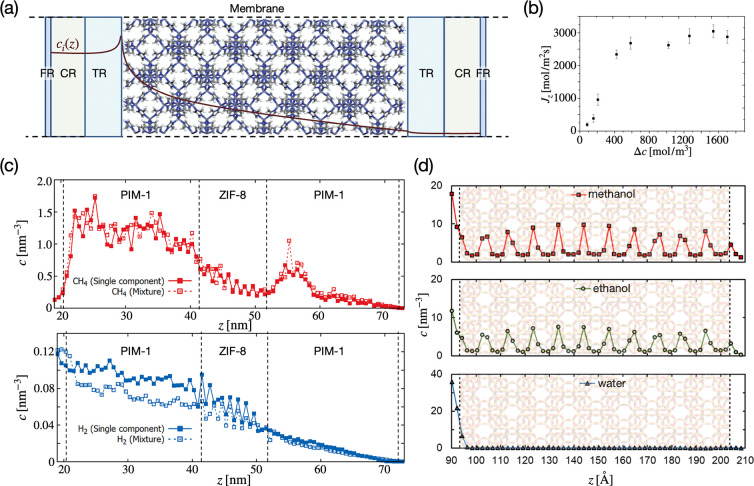
CμMD for permeation studies. (a) CGD-MD scheme: a concentration gradient is generated between the inlet (left) and the outlet (right) sides of a porous membrane. PBCs allow recirculating solute from the outlet to the inlet. (b) Variation of methane flux (*J*_*z*_) across a ZIF-8 membrane as a function of the concentration gradient *Δc*.^2^ (a) and (b) Adapted with permission from ref (2) (copyright 2017, the authors). Published by the Royal Society of Chemistry under a Creative Commons Attribution 3.0 Unported License. (c) Concentration profiles of methane (top) and hydrogen (bottom) across a composite PIM-1/ZIF-8 membrane in single-component and mixture simulations. Dashed lines indicate the interfaces of the PIM-1/ZIF-8 framework.^72^ Adapted with permission from ref (72) (copyright 2020 American Chemical Society). (d) Concentration profiles of methanol, ethanol, and water across an MFI membrane (in transparency), obtained via CGD-MD simulations.^73^ Adapted with permission from ref (73) (copyright 2021, Elsevier).

The first asymmetric implementation of CμMD, dubbed concentration-gradient-driven MD (CGD-MD),^2^ was applied to study the permeation of fluids and separation of mixtures through porous membranes. Herein, external forces given by eq 3 are applied asymmetrically at the inlet and at the outlet sides of a porous membrane (Figure 3a) to induce a constant concentration gradient across the membrane. As a result, the solute molecules flow through the membrane toward lower concentrations while PBCs allow solute recirculation and prevent depletion at the inlet, analogous to the dual control volume GCMD method.^41^ Herein, CμMD generates both a source and a sink of solute molecules to investigate the permeability and permselectivity of porous materials. By allowing accurate composition control of the gaseous/liquid phase at the inlet and outlet of a membrane (Figure 3a), via modulation of molecule recirculation across PBCs, the CGD-MD scheme enables the simulation of fluid mixture permeation and separation, without the necessity of particle insertion/deletion algorithms. This provides a valid alternative to the existing methodologies.^41,53,71^

Ozcan et al. applied CGD-MD to evaluate the permeabilities of methane, ethane, and ethylene across a ZIF-8 membrane. The approach demonstrated that the methane flux *J*_*z*_ across ZIF-8 strongly increases with the *Δc*, reaching a plateau beyond a threshold (Figure 3b), which separates transport control by concentration difference and mass transfer resistance. Remarkably, the CμMD-computed permselectivities of the ZIF-8 membrane for methane/ethane/ethylene separation follow the same trend of the experiments. While CGD-MD performs well when the target concentrations correspond to medium/high pressure, at low gas concentrations (∼1 atm) the instantaneous concentration in the CRs undergoes unavoidably large fluctuations, reducing the effectiveness of the method. This finite-size effect restricts the applicability range of CGD-MD as reported here; very large simulation volumes or reformulating the external forces could alleviate these issues.

Namsani et al.^74^ recently demonstrated that CGD-MD could be reliably used to simulate ternary mixture separation in porous membranes. Here, the separation of H_2_ in a ZIF-8 membrane from a H_2_, N_2_, and CO_2_ effluent syngas mixture, at the typical composition obtained from commercial production (air-blown autothermal reforming and water–gas shift reactions), was studied. The CGD-MD simulation at the feed temperature and pressure of a typical H_2_ purification unit (300 K and 35 atm) showed that the ZIF-8 membrane could select H_2_ over N_2_ and CO_2_ species, in agreement with experiments. These results contrasted with the widely used ideal permselectivity predictions, computed using self-diffusion coefficients and infinite dilution solubility factors. The study concluded that such approximate methods are inaccurate when mixtures of strongly associating gases or high-pressure ranges are considered. Methods that allow direct calculation of permeabilities, such as CGD-MD, are therefore crucial in these cases.

Ozcan et al. simulated the H_2_/CH_4_ transport and separation across the composite PIM-1/ZIF-8 membrane. The flux of H_2_ and CH_4_ gases and the H_2_/CH_4_ permselectivity in the composite PIM-1/ZIF-8 membrane (Figure 3c) were compared to results obtained for the individual PIM-1 and ZIF-8 membranes.^72^ The reported H_2_/CH_4_ permselectivity is reduced by ∼20% compared to the ideal permselectivity estimated via a macroscopic model^75^ using the data obtained for ZIF-8 and PIM-1 membranes separately. This demonstrated that macroscopic models, which compute the permeability of composite membranes by combining the permeabilities of individual components, can be inaccurate. These models do not account for interfacial effects and defects, such as nonselective void spaces present between the polymer and crystal membranes, which affect the permeability of the composite system. An approach such as CGD-MD, combined with an accurate description of the polymer/crystal interface, can directly quantify these interface-driven deviations from ideal permselectivty.

CGD-MD can also be used to study liquid-phase membrane separations. In ref (73), the permeation of alcohol/water mixtures through a silicalite (MFI) membrane was addressed via CμMD. Pure ethanol and methanol can diffuse through the membrane, while pure water diffusion is prevented (Figure 3d) due to hydrogen bonding with the interfacial silanol groups of MFI. When instead water/ethanol or methanol mixtures were tested, water permeation was observed. This is attributed to the competition of alcohol species against MFI silanol groups in forming hydrogen bonds with water, as the bonding of water with alcohol can enable the diffusion of both species across the silicalite membrane.

#### 2.2.2 “Cannibalistic” Crystallization

**Figure 4:**
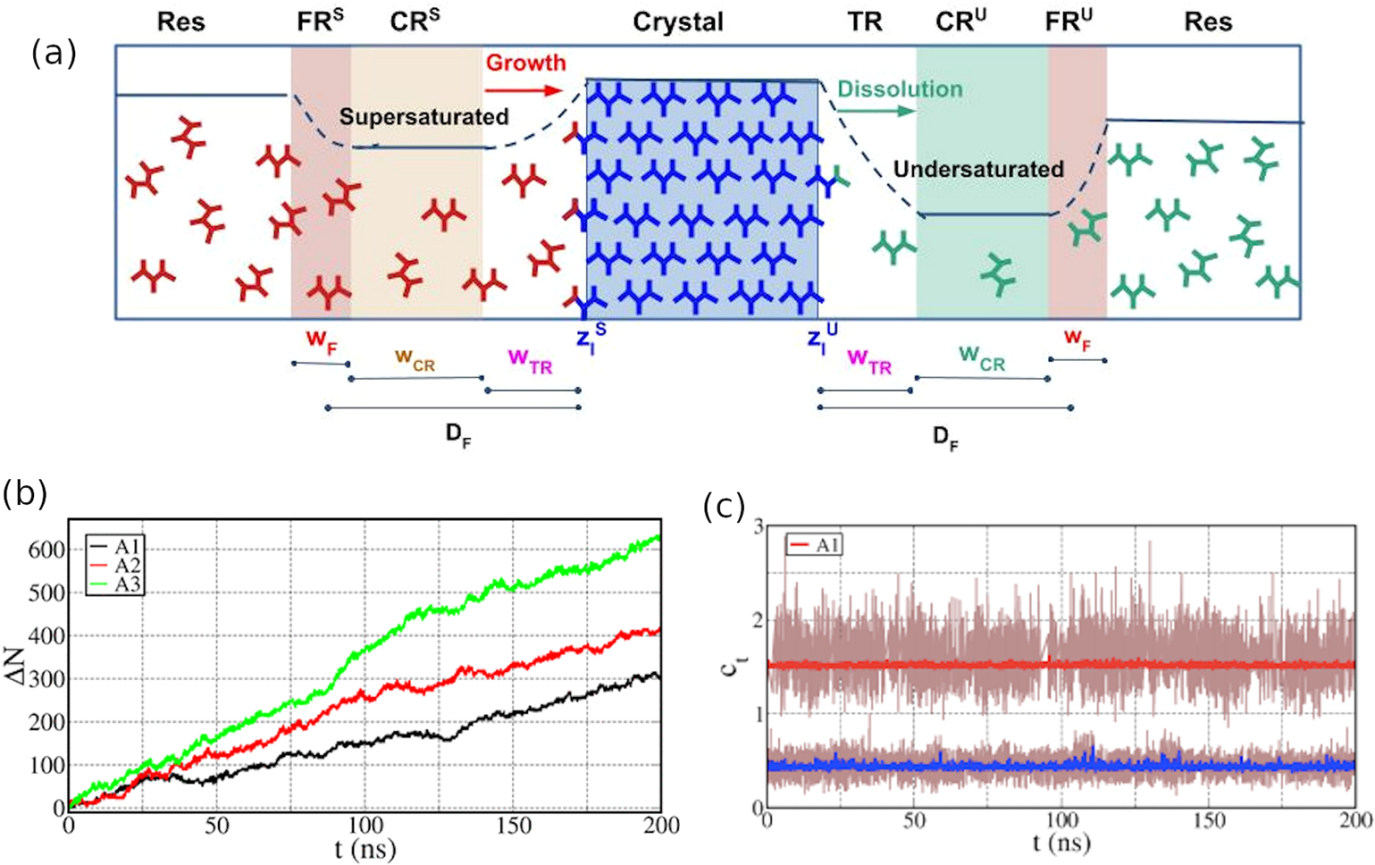
(a) Cannibalistic CμMD setup. The solution in the left CR is supersaturated (*S*), and in the right CR, it is undersaturated (*U*). The two crystal–solution interface positions, *z*_*I*_^*S*^ and *z*_*I*_^*U*^, are adaptively identified at every time step. (b) Growth profiles from three independent CμMD simulations at concentrations of 1.5, 2.0, and 2.4 nm^–3^. (c) Representative concentration profile: running averages demonstrate the effective control of the solution concentrations compared with target values. Adapted with permission from ref (70) (copyright 2018, American Chemical Society).

The asymmetric CμMD scheme was applied to simulate simultaneous growth and dissolution on the two opposite sides of a crystal slab, in an approach dubbed cannibalistic, referring to the feeding of the crystallization by means of the molecule of the very same crystallite. As done in the symmetric case (Figure 1c), also here a solid slab is exposed to two fluid phases; however, as in the CGD-MD scheme (Figure 3), the target concentrations of the two CR regions are different: one side of a crystal slab is exposed to a supersaturated solution leading to growth, while the other side is exposed to an undersaturated solution which favors the dissolution of the slab (Figure 4a).^70^ These two CR regions thus remove and supply solutes to the same reservoir region connected by PBCs. A proper balance between the growth and dissolution rates ensures that the reservoir can maintain the user-defined target concentrations irrespective of the total simulation time.

To check the effectiveness of this approach, we applied it to study the paradigmatic case of urea growth and dissolution in an aqueous solution.^70^ A series of independent CμMD simulations at different combinations of solution super- and undersaturation in the CRs were carried out.^70^ In each simulation, we observed multiple events of crystal layer growth and dissolution. The CR concentrations on both supersaturated and undersaturated sides remained close to their corresponding target values (Figure 4b,c) throughout the simulations, indicating that our method can maintain a constant chemical potential environment for an extended period of simulation. As a result, we could accurately calculate the growth and dissolution rates. It is worth noting that our cannibalistic method differs from the approach of Kusalik et al.^54,55^ in that CμMD implements solution concentration control, whereas temperature changes in direct forces are applied in that scheme.

### 2.3 Spherical Scheme: Nucleation

**Figure 5:**
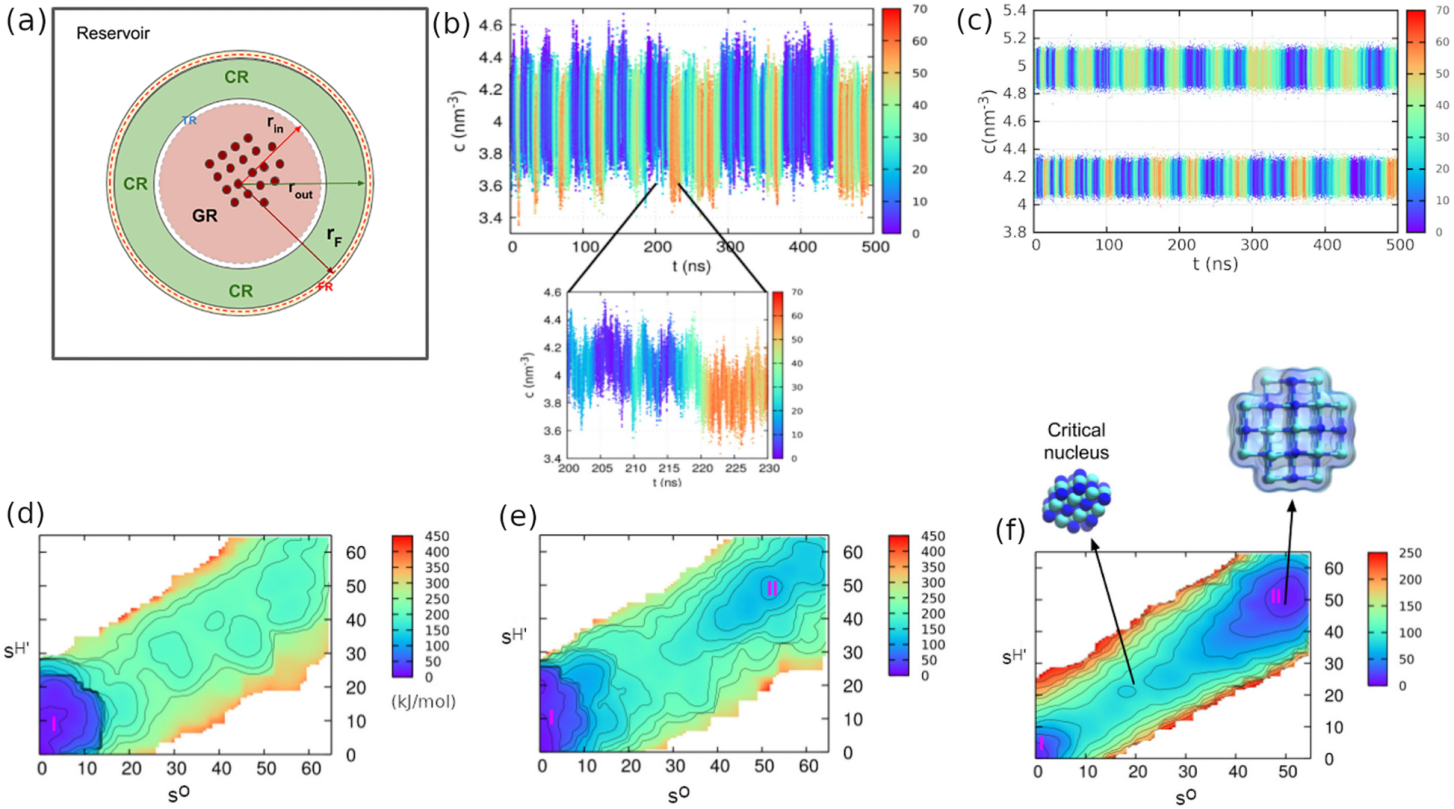
Spherical CμMD of NaCl nucleation from aqueous solution: (a) Spherical CμMD scheme. The GR is shown in pink, followed by a thin TR, and CR is shown in green. The FR surrounds the CR. The rest of the box acts as a molecular reservoir. A crystal nucleus is depicted in the GR (red spheres). (b) Solution concentration in the CR shell vs simulation time, obtained from standard NVT metadynamics, and (c) in the CμMD setup with two different target concentrations (upper and lower panels). The color indicates the nucleus size. The corresponding free-energy surfarces, as a function of the selected CVs (*s*^O^ and *s*^H'^), are depicted in (d)–(f); a representative critical nucleus and a rock-salt structure are shown in panel (f). Adapted with permission from ref (3). Copyright 2019 American Chemical Society.

Even more than in crystal growth, finite-size confinement effects have the potential to suppress nucleation by radically changing the thermodynamics of the process compared to a macroscopic open system.^9−14^ The idea of the CμMD simulation of crystallization has therefore been extended to the study of crystal nucleation. Unlike in the case of growth and adsorption simulations, in the case of nucleation, a more convenient spherical setup would allow the concentration to be controlled as a function of the radial distance (*r*_*i*_) from the center of nascent clusters of an emerging phase, in line with predictions (following classical nucleation theory) that nuclei are pseudospherical objects. In this setup, a cubic simulation box is considered. From its center, a spherical region is defined, which acts as the nucleus growth region (GR) (schematic in Figure 5a). Surrounding the GR, a set of concentric shells, which can be compared with the rectangular slabs in the planar CμMD setup (Figure 1), are defined to calculate the concentration of solute species (using eqs 2 and 3, the *z* coordinates should be replaced with *r*_*i*_). Similar to the planar model, spherical volume elements representing the transition region (TR), control region (CR), and force region (FR), respectively, are defined. The region beyond the FR is the molecular reservoir, which encapsulates the remaining region of the cubic cell with periodic boundary conditions applied in all directions.

This CμMD spherical variant was applied to investigate the nucleation of sodium chloride from an aqueous supersaturated solution.^3^ For comparison, one simulation in the standard NVT setup was carried out, followed by two CμMD simulations at two target solution concentrations, 4.2 nm^–3^ (molality 9.6 *m*) and 5.0 nm^–3^ (11.6 *m*). Note that the equilibrium solubility calculated using the Joung–Cheatham potential model^76^ is 3.7 *m*,^77,78^ and thus the two concentrations correspond to supersaturations, (*S* = *m*/*m*_*eq*_) 2.6 and 3.1, respectively.

Since nucleation involves high free-energy barriers at moderate supersaturation levels, WTMetaD was used in both the NVT and CμMD simulations to enhance the sampling of NaCl crystal states on the simulation time scale. Two CVs were used in the WTMetaD simulations. The first one (*s*^O^) was based on the environment-similarity CV developed by Piaggi and Parrinello,^79^ which describes the local ordering of atoms in a given crystal structure. The second CV (*s*^H'^) was the average number of water molecules surrounding Na^+^ ions within a given cutoff radius. Furthermore, these two CVs were modified to ensure that a nucleus forms inside the GR. Thanks to WTMetaD, multiple cycles of nucleation and dissolution were observed in the simulations. The concentration profiles obtained from a standard NVT (Figure 5a) and the two WTMetaD-CμMD simulations (Figure 5b) are shown. In the NVT simulation, each nucleation cycle is accompanied by a significant drop in the solution concentration in the CR. In contrast, the CR solution concentration in WTMetaD-CμMD simulations fluctuates around the target value during all crystal nucleation and dissolution events. Good control of the solution concentration during each nucleation cycle allowed us to accurately calculate nucleation free energies (Figure 5c–e) and to explicitly estimate supersaturation-dependent nucleation rates.^3^

## 3 Future Perspectives

We have herein summarized the main features and applications of the CμMD method. In the landscape of different techniques that have been proposed to implement concentration gradient simulations (Introduction section), CμMD has distinguished itself for its versatility and relatively facile application. The method takes advantage of the adaptive control of solution concentration, which provides a crucial vantage point in simulating the out-of-equilibrium evolution of many concentration-driven processes such as permeation, crystallization, adsorption, dissolution, etc. Originally developed to study crystal growth, CμMD has evolved into a multipurpose method. It is now regularly applied to study various physicochemical processes driven by concentration gradients beyond its original purpose, including nucleation and assembly, adsorption, membrane permeation, and separation. Following the applications discussed herein, CμMD could be applied to studying liquid–liquid transitions or the effect of solution environments (e.g., pH, reactant/product concentrations) on chemically reactive systems. Asymmetric CμMD generates a concentration gradient, enforcing the proper conditions to study permeation and crystal growth. This strategy can be adapted to probe concentration gradients typical of biological or electrochemical systems at nano/microscales and can be coupled with adaptive methods for the calculation of interfacial charges.^80^ While the spherical variant of CμMD is well-suited to investigate homogeneous nucleation in systems containing fast-diffusing solutes, this scheme could be easily adapted to probe the self-assembly of soft materials. As already demonstrated in crystallization studies, CμMD is easily coupled to enhanced sampling techniques such as Metadynamics, indicating a pathway for extending the validity range and potential of CμMD in future applications.

The future development of CμMD could also involve multiresolution schemes, such as the adaptive resolution technique of Kremer et al., allowing the simulation of very large systems with controlled compositions.^38^ Overall, CμMD applications are already wide-ranging, and exciting future developments will benefit simulation communities across multiple disciplines.

## Data Availability

The original CμMD method and its variants have been implemented in the open-source software PLUMED development versions. The CμMD code, input files, and related tutorials are available from the authors’ GitHub pages (https://github.com/mme-ucl/CmuMD,https://github.com/Tarakk/plumed-cumd), materials clouds archive (10.24435/materialscloud:2020.0015/v1 and 10.24435/materialscloud:2020.0013/v1,10.24435/materialscloud:k5-t2), and PLUMED-NEST^81^ portal (IDs). A tutorial on applying symmetric and asymmetric planar symmetry CμMD is available on the PLUMED website as a part of the 2022 PLUMED Masterclass (https://www.plumed.org/doc-master/user-doc/html/masterclass-22-8.html).

## Supporting Information Available

Theoretical discussion on concentration-driven processes and solution composition control (PDF)
