# Supplementary material for: Non-Equilibrium Modeling of Concentration-Driven processes with Constant Chemical Potential Molecular Dynamics Simulations
Source: Acc Chem Res. 2023 Apr 25;56(10):1156–67. doi: 10.1021/acs.accounts.2c00811 (PMC10193523; doi:10.1021/acs.accounts.2c00811)
Supplement: Supplementary file 1 — ar2c00811_si_001.pdf [file ar2c00811_si_001.pdf]

Supporting Information

Non-Equilibrium Modelling of Concentration-Driven processes with Constant Chemical Potential Molecular Dynamics Simulations

Tarak Karmakar,[*,†] Aaron R. Finney,[‡] Matteo Salvalaglio,[*,‡] A. Ozgur Yazaydin,[‡] and Claudio Perego[*,¶]

†*Department of Chemistry, Indian Institute of Technology, Delhi, Hauz Khas, New Delhi 110016, India*

‡*Dept of Chemical Engineering, University College London, Torrington Place, London, WC1E 7JE*

¶*Department of Innovative Technologies, University of Applied Sciences and Arts of Southern Switzerland, Polo Universitario Lugano, via la Santa 1, 6962, Lugano-Viganello, Switzerland*

E-mail: tkarmakar@chemistry.iitd.ac.in; m.salvalaglio@ucl.ac.uk; claudio.perego@supsi.ch

Constant driving force from controlled composition

In what follows, we discuss the foundations of the core idea of the CμMD method. In particular we discuss how regulating the solution composition allows establishing a constant driving force for concentration-driven, out-of-equilibrium processes.

The idea behind controlling the driving force of concentration-driven processes builds on the definition of chemical potential in multicomponent fluids. Here we briefly review how and under which set of hypotheses controlling the composition of a liquid phase leads to controlling its chemical potential and, thus, alongside, the driving force associated with non-equilibrium concentration-driven processes. The chemical potential of component i in a multi-component liquid phase can be defined as:

$$\mu_i^\ell = \mu_0 + k_B T \log\left(\gamma_i\left(\mathbf{x}, T, P\right) x_i\right) \tag{1}$$

where $\mathbf{x} = (x_1, \ldots, x_N)$ is the liquid phase mole fraction vector, x_i is the mole fraction of component i, γ_i is the activity coefficient of component i, defined as the ratio between its fugacity in a real mixture at composition $\mathbf{x}$ and in an ideal mixture at the same composition, and μ_0 the chemical potential of pure i, chosen as the reference state. N is the total number of chemical species. The molar fraction of component i is defined as the ratio c_i/c_{tot}, where c_i is the molar concentration of component i, and c_{tot} is the total molar concentration of the liquid phase. The product $\gamma_i(\mathbf{x}, T, P)x_i$ gives the activity $a_i(\mathbf{x}, T, P)$ used in Eq. 1 in the main manuscript to embed the composition dependence of the mass transfer driving force.

By analyzing Eq. 1, one can readily observe that by maintaining constant the concentration of $N-1$ arbitrary components in the liquid phase $\mathbf{x}$, and thus μ_i^ℓ, remain constant. As discussed in the later paragraphs, CμMD enables controlling the concentration of at least one component in selected regions of the simulation box where said component can be found in the fluid phase, and where the behaviour of a bulk multicomponent fluid phase is recovered. It can be readily seen that for a binary liquid phase where $\mathbf{x} = c_{tot}^{-1}\left[c_i, c_{tot} - c_i\right]$, maintaining

constant the concentration of i is sufficient to keep μ_i^ℓ constant.

When a fluid phase of interest, characterized by chemical potential μ_i^ℓ is in contact with an "active region", such as a phase boundary or a solid/liquid interface, acting either as a sink or a source of i at constant chemical potential, a mass transfer to/from the active region is established. In these conditions, controlling c_i in the liquid phase enables controlling the thermodynamic driving force associated with mass transfer to/from the active region.

As an example, a sink/source of i molecules at constant μ could be a crystal surface of pure i at constant T and P. In this case, the chemical potential of the crystal at T and P will be defined as:

$$\mu_i^{xtal} = \mu_0 + k_B T \log\left(\gamma_i\left(\mathbf{x}_{eq}, T, P\right) x_{i,eq}(T, P)\right) \tag{2}$$

where $x_{i,eq}(T, P)$ is the solubility of i, a constant at T and P assigned.

In these conditions, the driving force associated with a displacement of i across the phase boundary becomes a function of the solution composition:

$$\Delta\mu^{\ell \to xtal} = -k_B T \log\left(\frac{\gamma_i\left(\mathbf{x}, T, P\right) c_{tot}^{-1} c_i}{\gamma_i\left(\mathbf{x}_{eq}, T, P\right) x_{i,eq}(T, P)}\right) \tag{3}$$

By controlling $N - 1$ concentrations with CμMD (i.e. just the concentration of i in a two-component system) $\Delta\mu^{\ell \to xtal}$ enables a control of the mass transfer driving force. Simulations evolving in these conditions would capture the dynamics of out-of-equilibrium mass transfer processes, under the effect of a constant driving force, for example, mimicking experiments where crystal faces grow under the effect of constant supersaturation. Moreover, in these conditions, the concentration and chemical potential gradients established between the sink/source and the liquid phase under controlled composition can be interpreted within mesoscale, continuous models of mass transfer.
